# Contribution of Mesenchymal-like and Epithelial Cellular Subsets to Chemotherapy Resistance in Triple-Negative Breast Cancer

**DOI:** 10.3390/ijms27073157

**Published:** 2026-03-31

**Authors:** Ngoc B. Vuong, Olga Y. Korolkova, Michael G. Izban, Nobelle I. Sakwe, Antonisha R. McIntosh, Destiny D. Ball, Perrin J. Black, Alayjha D. Edwards, Billy R. Ballard, Samuel E. Adunyah, Amos M. Sakwe

**Affiliations:** 1 Department of Biomedical Sciences, School of Graduate Studies, Meharry Medical College, Nashville, TN 37208, USA; 2 Department of Pathology, School of Medicine, Meharry Medical College, Nashville, TN 37208, USA; 3 Department of Biochemistry Cancer Biology, Neuroscience and Pharmacology, School of Medicine, Meharry Medical College, Nashville, TN 37208, USA

**Keywords:** triple-negative breast cancer, chemotherapy, mesenchymal-like, epithelial, Annexin A6, Ki67, Vimentin, drug resistance, p90 ribosomal S6 Kinases

## Abstract

Triple-negative breast cancer (TNBC) tumors are typically heterogeneous, predominantly epithelial tissues with discrete patches of mesenchymal-like TNBC cells that differ in their invasiveness, proliferation potential and response to treatment. However, the impact of mesenchymal-like and epithelial TNBC cells on the persistence of chemotherapy-resistant disease remains poorly understood. Mesenchymal-like and epithelial TNBC cell types were detected by multiplex fluorescent immunohistochemistry using antibodies against vimentin, Ki67, and Annexin A6 (AnxA6). Chemotherapy drug-resistant mesenchymal-like and epithelial TNBC cell populations were established by pulse exposure and stepwise dose escalation and validated by 3D cultures and unbiased antibody arrays. Analysis of the response of TNBC tumors treated with six common chemotherapy regimens resulted in 36% complete response and 64% partial response with residual tumor sizes ranging from 0.5 to 37.0 mm. Treatment of TNBC cells with chemotherapy agents led to distinct resistance signatures including downregulation of survivin and upregulation of M-CSF and CXCL8/IL-8 in the model mesenchymal-like TNBC cells, and upregulation of CCL2/MCP-1, CTSS and DKK-1 in model epithelial TNBC cells. The inhibitory phosphorylation of GSK-3β (p-S9) increased in paclitaxel-resistant epithelial cells but decreased in resistant mesenchymal-like TNBC cells. Finally, chemotherapy resistance also activated p90 ribosomal S6 kinases (RSK1/2) in both cell types, while activation of mitogen- and stress-activated kinases (MSK1/2) was only observed in chemotherapy-resistant epithelial TNBC cells. These data reveal that chemotherapy resistance of epithelial and mesenchymal-like TNBC cellular subsets led to distinct profiles of proinflammatory and immune cell chemotactic cytokines and modulated the activities of GSK-3β, p90 RSK1/2 and the related MSK1/2. Targeting these factors and/or the associated signaling pathways may help overcome chemotherapy resistance in TNBC.

## 1. Introduction

Triple-negative breast cancer (TNBC) represents 15–20% of incident breast cancers, but accounts for nearly 40% mortality within the first 5 years after diagnosis, compared to other breast cancer subtypes [1]. This highly aggressive breast cancer subtype is characterized by the absence of estrogen and progesterone receptors and HER2 amplification. The lack of these receptor makes TNBC tumors particularly challenging to treat, as they do not respond to either hormonal therapies or HER2-targeted treatments [2]. Consequently, chemotherapy remains the treatment of choice for TNBC, and despite initial responsiveness, TNBC rapidly relapses and becomes resistant to subsequent treatments [3,4,5].

Many studies have used gene expression profiling to demonstrate that the diverse response of TNBC tumors to treatment is partly due to tumor heterogeneity. This includes distinct TNBC molecular subtypes which now comprise mesenchymal-like (MSL), basal-like immune activated (BL1/BLIA), basal-like immune suppressed (BL2/BLIS), and luminal androgen receptor positive (LAR) [6,7,8,9]. These TNBC molecular subtypes are also known to exhibit overlapping alterations in DNA repair pathways, metabolic pathways, immune checkpoint components as well as significant differences in the composition of the tumor microenvironment [10]. Although these classifications provide insight into personalized treatment options, the overlapping alterations invariably contribute to the varied responses of TNBC tumors to chemotherapy and subsequent development of resistance [11]. Therefore, the impact of these classifications on clinical outcomes remains unpredictable.

The complexity of TNBC tumors, like most breast cancers, also includes multiple breast cancer subtypes [8,9,10,11,12] and morphologically distinct mesenchymal-like and epithelial cell types that exhibit distinct biological behaviors including invasiveness and therapeutic susceptibilities [13,14,15]. As the disease progresses, the impact of intra-tumoral heterogeneity on treatment resistance is partly defined by evolution of distinct phenotypic cell populations [16,17,18,19]. Treatment resistance is also defined by how the cellular subsets, including heterogeneous cancer stem cell populations, with distinct intrinsic drug sensitivities co-exist within the same tumor [20,21]. Therefore, a better understanding of the heterogeneity and plasticity of tumor cells may lead to the development of more effective therapeutic strategies [22]. Epithelial TNBC cells typically retain cell–cell adhesion properties and lead to larger but less invasive tumors. Mesenchymal-like TNBC cells, on the other hand, are characterized by their enhanced migratory and invasive properties, often associated with epithelial-to-mesenchymal transition (EMT), stem cell-like properties and resistance to apoptosis [23,24]. Given that typical TNBC tumors consist of distinct proportions of epithelial cells and those at various stages of EMT, a better understanding of subtle molecular differences between these phenotypically distinct TNBC cell types following chemotherapy is essential to eventually develop novel and more effective treatment regimens.

The impact of phenotypic diversity measured by Simpson’s score does not always correlate with genetic intra-tumor heterogeneity measured by the MATH index, especially on responses to treatment [25]. Classification of TNBC into proliferative basal-like (herein denoted as epithelial) and the more invasive mesenchymal-like subsets relies on cell morphology and expression of phenotypic molecular markers such as the epithelial E-cadherin and the mesenchymal vimentin [2,23,26]. Both markers are known to be heterogeneously expressed and change with cancer progression, requiring the use of other cell proliferation markers such as Ki67, or other markers of invasiveness. Detection of Ki67 and Annexin A6 (AnxA6), a multifunctional scaffolding protein that is critical in cell proliferation, survival, migration, membrane repair and drug resistance [27,28,29,30] has previously been reported to delineate AnxA6-high invasive from AnxA6-low rapidly growing TNBC cell lines and patient-derived xenograft models [31]. While this supports the detection of AnxA6 in TNBC tumors as a marker of invasiveness, its association with the expression of vimentin and resistance to chemotherapy in TNBC cells remains unclear.

The goal of this study was to identify potential mechanistic differences on the survival of mesenchymal-like and epithelial TNBC cellular subsets during chemotherapy resistance. We assessed the response of TNBC patients to six different chemotherapy regimens, established the detection and distribution of epithelial and mesenchymal-like cells within the tumors, and identified molecular differences between chemotherapy-resistant, phenotypically distinct mesenchymal-like and epithelial TNBC cell types. These data reveal that chemotherapy resistance of epithelial and mesenchymal-like TNBC cellular subsets led to distinct profiles of proinflammatory and immune cell chemotactic cytokines and modulated the activities of GSK-3β, p90 RSK1/2 and the related MSK1/2. Targeting these factors and/or the associated signaling pathways may help overcome chemotherapy resistance in TNBC.

## 2. Results

### 2.1. AnxA6 and Ki67 Expression Defined Mesenchymal-like and Epithelial Phenotypes in TNBC Tumors

**Figure 1:**
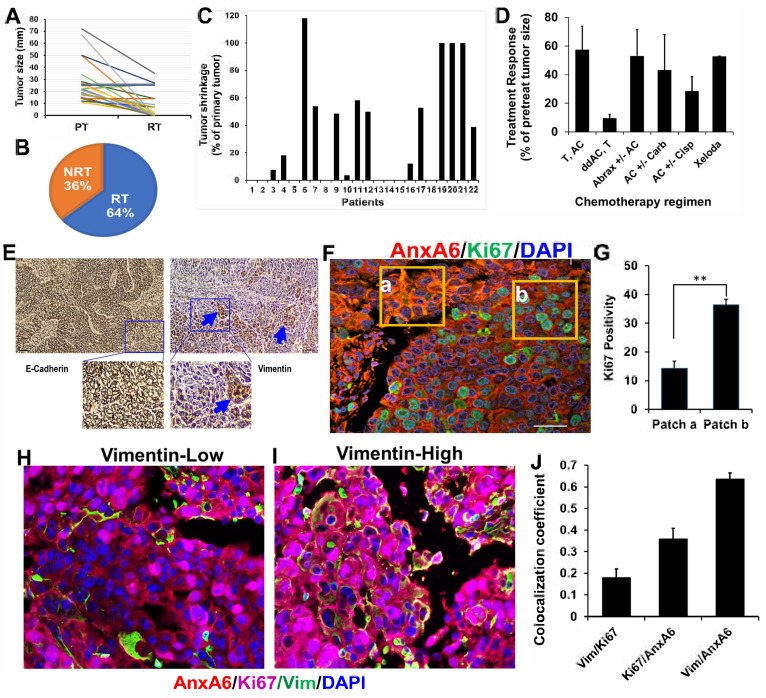
**Response of stage IV TNBC tumors to different standard of care chemotherapy regimens and detection of epithelial and mesenchymal-like tumor cells**. (**A**–**D**) Analysis of the response and tumor sizes in TNBC patients before and after six different neoadjuvant chemotherapy regimens. NRT = No Residual Tumor; RT = Residual Tumor; T = Taxol; AC = Adriamycin and Cytoxan; Cisp = Cisplatin; Carb = Carboplatin; ddAC = dose-dense Adriamycin; Abrax = Abraxane; Xeloda = Capecitabine. (**E**) Detection of E-cadherin and vimentin patches (blue arrows) in a typical TNBC tumor by immunohistochemistry (IHC). (**F**,**G**) Validation of the inverse expression of AnxA6 (red), and cell proliferation marker Ki67 (green) in TNBC patient tissues and semi-quantification of the fluorescent intensity of Ki67 positivity. Nuclei are stained blue. (**H**–**J**) Relationship between AnxA6, Ki67 and vimentin in a typical TNBC tumor by multiplex fluorescent IHC. All images were taken at 20× magnification. ** denotes *p* < 0.01.

We assessed the clinical response of TNBC patients previously described in Korolkova et al., 2020 [31] to six typical neoadjuvant chemotherapy combinations of Doxorubicin (Adriamycin), cyclophosphamide (Cytoxan), platinum-based cytotoxic compounds (cisplatin, carboplatin), abraxane (nab-paclitaxel), paclitaxel (Taxol), and capecitabine (Xeloda). A review of the clinical data for the 22 patients indicates that 8 patients (36%) were black with an average age at diagnosis of 52.5 years, and 14 patients (64%) were white with an average age at diagnosis of 52 years. Two patients had primary tumors with about seven satellite lesions and only two patients were diagnosed with lung, liver, and/or brain metastases. Out of 22 patients, 8 (36%) displayed complete response with no residual tumors (NRT), and 14 (64%) displayed progressive or stable disease or had residual tumors (RT) with tumor sizes varying from 0.5 to 35 mm (Figure 1A–C). The average age at diagnosis for patients with no residual tumors was 53.4 years (*n* = 8) while those with residual tumor was 51 years (*n* = 14). Out of the six regimens, dense dose anthracycline/Cytoxan (AC) followed by taxol (ddAC,T) was the most effective at reducing tumor size. Combinations of AC and T were the least effective, with larger recurrent tumors (Figure 1D).

To gain a better understanding of the diverse response to these treatment regimens, we assessed expression of E-cadherin and vimentin by IHC. This showed that a typical TNBC tumor is a predominantly epithelial tissue with discrete patches of mesenchymal-like TNBC cells (Figure 1E). This phenotype was further confirmed by double fluorescent IHC of the proliferation marker Ki67 and AnxA6, a calcium-dependent membrane binding protein with high expression in invasive TNBC cells and low expression in proliferative TNBC cells [31]. There were discrete tumor patches, e.g., tumor area a with high expression of AnxA6 and low expression of Ki67 (AnxA6hi/Ki67lo) with ~14% Ki67 positivity, while the bulk of the tumor represented by tumor area b showed AnxA6lo/Ki67hi with >36% Ki67 positivity (Figure 1F,G). Meanwhile, vimentin staining was dispersed in vimentin-low patches and intensely expressed in more cells in vimentin high areas (Figure 1H,I). In a typical TNBC tumor, the probability for co-expression of AnxA6 and vimentin is higher than with Ki67 or between AnxA6 and Ki67 (Figure 1J). These data suggest that detection of Ki67, vimentin and AnxA6 can be used to assess phenotypic tumor heterogeneity and confirms that typical TNBC tumors are predominantly epithelial tissues with discrete areas of mesenchymal-like tumor cells.

**Figure 2:**
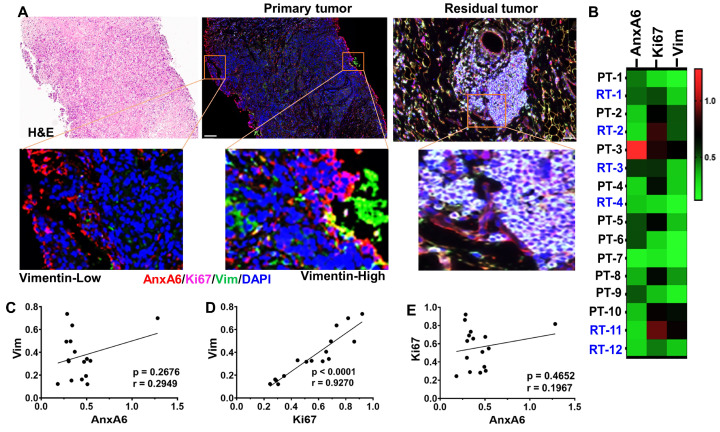
**Association of AnxA6 and Ki67 expression with vimentin expression in pre-treatment biopsies and chemotherapy residual disease tumors**. (**A**,**B**) Expression of AnxA6 (red), Ki67 (purple) and Vimentin (green) in primary tumor versus in residual tumor tissues. (**A**) Tissues were stained with H&E and by using tyramide-based fluorescent IHC using antibodies against AnxA6 (red), Ki67 (far red), vimentin (green) and cell nuclei were stained with DAPI (blue). Images were captured at 20× magnification. (**B**) Heatmap showing the relative expression of AnxA6, Ki67 and vimentin in selected biopsy (PT) and residual tumor (RT) quantified using the Ariol software (*n* = 16). (**C**–**E**) Analysis of the correlation in expression between Ki67, AnxA6 and vimentin; r denotes the correlation coefficient, *p* denotes statistical significance for each comparison.

We next assessed whether expression of AnxA6 and Ki67 is associated with vimentin expression in pre-treatment biopsies and chemotherapy residual disease tumors by multiplex immunofluorescence (Figure 2A). The expression levels of these proteins in these tissues is shown as a heatmap (Figure 2B). Analyses of the co-expression of these proteins confirmed the reciprocal expression of AnxA6 and Ki67 in all tissues tested as previously reported [31]. Although co-expression of vimentin and AnxA6 was observed in some but not all tissues, expression of vimentin in both primary and chemotherapy residual tumors is strongly correlated with Ki67 (r = 0.9270, *p* = 0.0001), but not with AnxA6 (r = 0.1967, *p* = 0.4652). Despite the reciprocal expression of AnxA6 and Ki67, the expression of AnxA6 is not significantly correlated with that of Ki67 (Figure 2C–E).

### 2.2. Prototype Epithelial and Mesenchymal-like TNBC Cell Lines Display Distinct Susceptibility to Standard of Care Chemotherapy Agents

As demonstrated in previous studies [32,33,34], the vimentin expressing BT-549 (mesenchymal-like model) TNBC cells express relatively high levels of AnxA6 and low levels of Ki67 positivity (Supplementary Figure S1A,C), while the E-cadherin positive MDA-MB-468 (epithelial or basal-like model) TNBC cells express relatively low levels of AnxA6 and correspondingly, high levels of Ki67 positivity (Supplementary Figure S1B,C). To validate this reciprocal expression of AnxA6 and Ki67, we also show that EdU incorporation in MDA-MB-468 cells was >3-fold compared to that in BT-549 cells (Supplementary Figure S3D–F).

We next assessed the response of these model mesenchymal-like and epithelial TNBC cells to the cytotoxic effects of doxorubicin (DOX), paclitaxel (PTX) and carboplatin (CBP). Treatment of the parental cells with various concentrations of these drugs for 72 h revealed that the epithelial MDA-MB-468 cells were 2 to 7-fold more sensitive to these drugs compared to the mesenchymal-like BT-549 cells (Supplementary Figure S1G–I). Thus, BT-549 and MDA-MB-468 cell lines are not only phenotypically distinct but are also physiologically distinct in their proliferation rates and response to standard-of-care chemotherapy agents.

We next established chemotherapy drug-resistant populations of the mesenchymal-like BT-549 and the epithelial MDA-MB-468 TNBC cells by pulse exposure and stepwise dose escalation as described in Section 4.2. Consistent with the differential response of these cells to the three chemotherapy drugs, the maximal in vitro tolerated concentrations for BT-549 cells (100 nM DOX, 30 nM PTX, and 70 µm CBP) were ~2-fold higher than those for MDA-MB-468 cells (50 nM DOX, 15 nM PTX and 40 µM CBP). The IC50 values and fold resistance to these drugs revealed that BT-549 cells develop resistance to CBP and DOX but are refractory to PTX (Supplementary Figure S2A–C, Table 1), while MDA-MB-468 cells effectively developed resistance to all three chemotherapy agents (Supplementary Figure S2D–F, Table 1).

**Figure 3:**
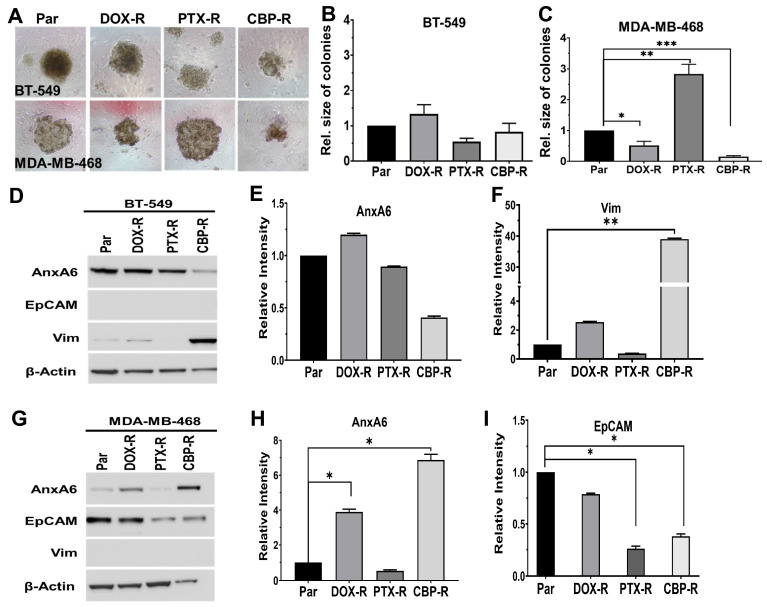
**Phenotypic shift in chemotherapy-resistant TNBC cells from AnxA6 and either vimentin or EpCAM in TNBC cells.** (**A**–**C**) Resistant mesenchymal-like BT-549 and epithelial MDA-MB-468 cells were cultured in ultra-low attachment plates for up to 10 days in medium containing the maintenance concentrations of each drug. Digital images of colonies captured at 10× magnification (**A**) and quantification of the size of the colonies or cell masses (*n* > 10) using ImageJ version 1.54d for BT-549 (**B**) and MDA-MB-468 (**C**). (**D**,**G**) Cell lysates from parental and drug-resistant BT-549 (**D**) and MDA-MB-468 (**G**) cells were prepared as described in Section 4.2, and the expression of AnxA6, EpCAM, and vimentin was determined by Western blotting. β-actin was used as the loading control. (**E**,**F**,**H**,**I**) Densitometric analysis of protein bands using ImageJ for AnxA6 (**E**) and vimentin (**F**) in BT-549 cells and for AnxA6 (**H**) and EpCAM (**I**) in MDA-MB-468 cells relative to control cells from three independent experiments. * denotes *p* < 0.05; ** denotes *p* < 0.01; *** denotes *p* < 0.001.

To confirm the differential response of these cell types to these chemotherapy agents, we compared the growth of chemotherapy drug-resistant cells to untreated parental control cells cultured in 3D cultures in ultra-low attachment plates. In agreement with the dose response curves (Supplementary Figure S2), the growth of chemotherapy-resistant BT-549 cells was not different from that of parental cells (Figure 3A,B). However, for MDA-MB-468 cells, PTX-R cells grew ~3-fold faster, while DOX-R and CBP-R cells grew slower than parental cells (Figure 3A,C). Therefore, although epithelial cells are more sensitive to these chemotherapy agents, this cell type has a higher propensity to develop resistance to any of these drugs.

### 2.3. Differential Expression of AnxA6 and Markers of Invasiveness Defined Chemotherapy-Resistant Phenotypically Distinct TNBC Cells

We next assessed how resistance to these drugs affected the expression of AnxA6, Ki67 and vimentin, using parental untreated cells as controls. By immunofluorescence of BT-549 cells, AnxA6 was upregulated in DOX-R, unchanged in PTX-R and strongly downregulated in CBP-R cells (Supplementary Figure S3A,B). Ki67 expression decreased in both DOX-R and PTX-R but was more intense in CBP-R cells compared to parental BT-549 cells (Supplementary Figure S3A,C). Vimentin on the other hand increased in PTX-R and CBP-R cells but was unchanged in DOX-R cells (Supplementary Figure S3A,D). In MDA-MB-468 cells, AnxA6 expression was upregulated by 4- to 10-fold in the DOX-R, PTX-R and CBP-R cells (Supplementary Figure S3E,F), while the expression of Ki67 decreased in PTX-R and CBP-R but tended to increase in DOX-R cells compared to the parental cells (Supplementary Figure S3E,G). Vimentin expression also strongly increased in the resistant cells (Supplementary Figure S3E,H).

To further define the shift in the phenotype of chemotherapy-resistant TNBC cells, we assessed the expression of vimentin and AnxA6 by Western blotting using whole cell lysates from parental and drug-resistant cells. In CBP-R BT-549 cells, the expression of AnxA6 was downregulated while vimentin expression was upregulated (Figure 3D–F). Meanwhile, in MDA-MB-468 cells, AnxA6 expression increased in DOX-R and CBP-R cells but strongly decreased in PTX-R cells (Figure 3G,H). Expression of epithelial cell adhesion molecule (EpCAM) was also strongly decreased in PTX-R and CBP-R cells and unchanged in DOX-R cells (Figure 3G,I). Consistent with heterogeneous response to various chemotherapy regimens depicted in Figure 1, PTX and CBP treatment induced opposite effects in the expression of AnxA6, vimentin and Ki67 albeit in a cell-type-dependent manner. These data indicate that the three standard-of-care chemotherapy drugs induce profound phenotypic changes, particularly in epithelial cells that potentially contribute to the differential susceptibility of these cell types to chemotherapy.

### 2.4. Chemotherapy-Resistant Mesenchymal-like and Epithelial TNBC Cellular Subsets Exhibit Distinct Signaling Protein Expression Patterns

**Figure 4:**
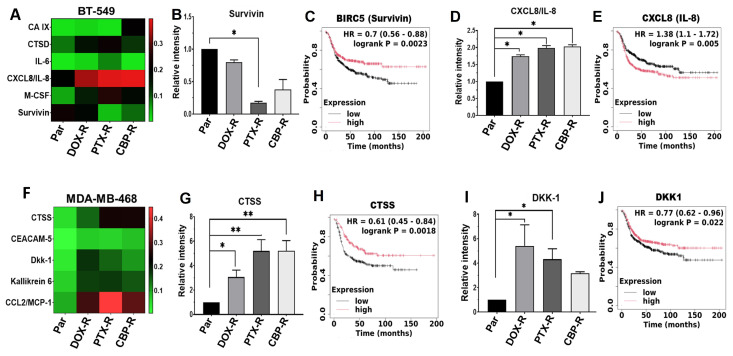
**Pattern of differently expressed proteins in chemotherapeutic resistant mesenchymal-like and epithelial TNBC cells**. (**A**,**F**) Heatmaps showing the differentially expressed malignancy promoting proteins in cell lysates from chemotherapy-resistant BT-549 (**A**) and MDA-MB-468 (**F**). Detected proteins on replica dot blots were quantified using ImageJ software and normalized to internal controls. (**B**–**E**) Densitometric analysis of protein dots (**B**,**D**) from three independent experiments, and the corresponding Kaplan–Meier (KM) plots (**C**,**E**) for survivin (**B**,**C**) and interleukin 8 (**D**,**E**) in BT-549 cells. (**G**–**J**) Densitometric analysis of protein dots (**G**,**I**) and the corresponding Kaplan–Meier (Km) plots (**H**,**J**) for CTSS (**G**,**H**) and DKK1 (**I**,**J**) in MDA-MB-468 cells. Indicated in the KM plots is the relationship between high (red) or low (black) gene expression of the indicated genes and the survival of basal-like breast cancer patients, the hazard ratio (HR) and statistical significance. * denotes *p* < 0.05; ** denotes *p* < 0.01.

To gain a better understanding of how chemotherapy resistance is sustained in epithelial and mesenchymal-like TNBC cells, we profiled the expression of 84 cancer associated proteins in cell lysates from parental control and drug-resistant BT-549 and MDA-MB-468 TNBC cells using the human oncology antibody array kit (R&D system). We identified six differentially expressed proteins in resistant mesenchymal-like BT-549 cells: carbonic anhydrase IX (CAIX), cathepsin D (CTSD), interleukin 6 (IL-6), CXCL8/IL-8, macrophage colony-stimulating factor (M-CSF), and the inhibitor of apoptosis survivin (Figure 4A). CXCL8/IL-8 and M-CSF were the most upregulated in DOX, CBP, or PTX-resistant mesenchymal-like cells. Correspondingly, the cellular levels of survivin were remarkably downregulated in PTX-R and CBP-R cells than in DOX-R cells (Figure 4A). Since most triple-negative cancers are basal-like and the majority of tumors designated as basal-like are triple-negative [35,36], we sought to determine if these genes significantly influenced the survival of patients with basal-like breast cancer using the publicly available KM Plotter tool. This analysis revealed that among the six differentially expressed proteins, low expression of survivin (Figure 4B,C), and high expression of CXCL8/IL-8 (Figure 4D,E) were significantly associated with poor relapse free survival (RFS) of basal-like breast cancer patients.

In the epithelial MDA-MB-468 cells, resistance to chemotherapy agents led to upregulation of monocyte chemotactic protein (CCL2/MCP-1), cathepsin S (CTSS), Dickkopf WNT signaling pathway inhibitor 1 (DKK-1), and Kallikrein-related peptidase 6 (KLK6) compared to parental cells (Figure 4F). As in BT-549 cells, the expression levels of these proteins were differentially influenced by the three chemotherapy agents and CCL2/MCP-1 was the most upregulated in all three drug-treated cells. Patient survival analysis using the KM Plotter database revealed that reduced expression of cathepsin S (CTSS) (Figure 4G,H) and DKK-1 are significantly associated with poor RFS of basal-like breast cancer patients (Figure 4I,J).

**Figure 5:**
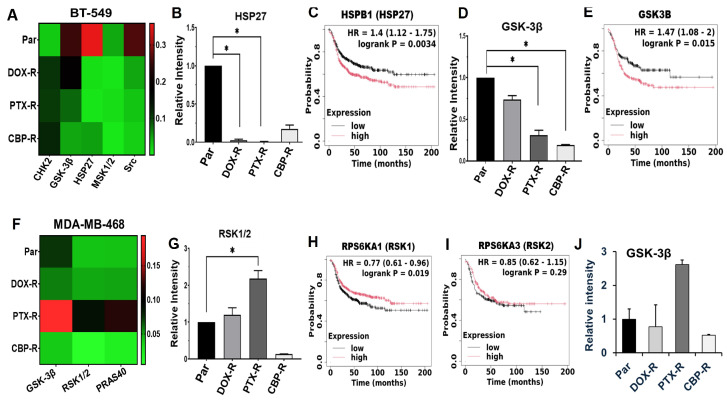
**Identification of differently expressed activated protein kinases in chemotherapy-resistant mesenchymal-like and epithelial TNBC cells**. (**A**,**F**) Heatmaps showing the differentially phosphorylated malignancy promoting kinases in cell lysates from chemotherapy-resistant BT-549 (**A**) and MDA-MB-468 (**F**). Detected phospho-kinases on replica dot blots were quantified using ImageJ software version 1.54d and normalized to internal controls. (**B**–**E**) Densitometric analysis of phosphoprotein dots (**B**,**D**) and Kaplan–Meier (KM) plots (**C**,**E**) for HSP27 (**B**,**C**) and GSK-3β (**D**,**E**) in BT-549 cells. (**G**–**J**) Densitometric analysis of phosphoprotein dots (**G**,**J**) from three independent experiments, and Kaplan–Meier (Km) plots (**H**,**I**) for RSK1/2 (**G**–**I**) and GSK-3β (**E**,**J**) in MDA-MB-468 cells. Indicated in the KM plots is the relationship between high (red) or low (black) gene expression of the indicated genes and the survival of basal-like breast cancer patients, the hazard ratio (HR) and statistical significance. * denotes *p* < 0.05.

To identify signaling pathways that potentially help maintain chemotherapy resistance in the phenotypically distinct TNBC cell subsets, we profiled 37 activated protein kinases using the human phospho-kinase antibody array kit (R&D Systems). In the mesenchymal-like BT-549 cells, phosphorylation of Glycogen synthase kinase-3 beta (GSK-3β) at p-S9, Heat shock protein 27 (HSP27) at p-S78/S82, MSK1/2 (p-S376/360) and the non-receptor tyrosine kinase Pp60c-Src (Src) at p-Y419 were decreased, while the phosphorylation of Checkpoint Kinase 2 (CHK-2) at p-T68 increased in chemotherapy agent-resistant cells compared to the control parental BT-549 cells (Figure 5A). To test whether these proteins are clinically relevant factors in basal-like breast cancer, we show that high expression of HSP27 (Figure 5B,C) and GSK-3β (Figure 5D,E) is associated with poor survival of basal-like breast cancer patients. We also show that in the epithelial MDA-MB-468 cells, phosphorylation of GSK-3β (p-S9), p90 ribosomal S6 kinases (RSK1/2) at p-S221/S227, and Proline-rich Akt substrate 40 kDa (PRAS40) at p-T246 were remarkably increased in the PTX-R cells compared to DOX-R and CBP-R cells (Figure 5F,G,J).

**Figure 6:**
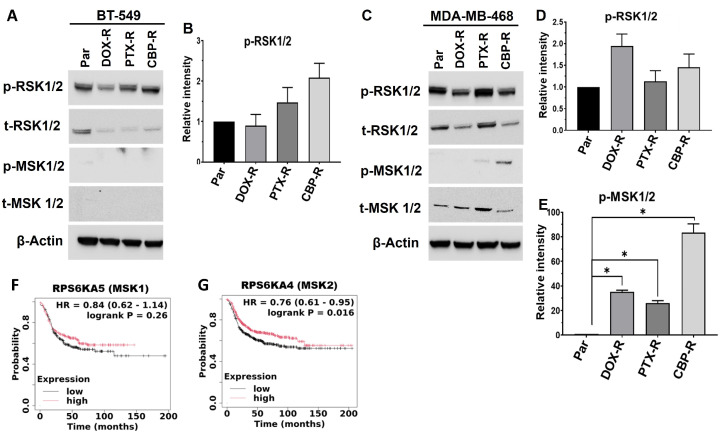
**Validation of RSK1/2 and MSK1/2 phosphorylation in chemotherapy-resistant BT-549 and MDA-MB-468 cells**. (**A**,**C**) Total and phosphorylated levels of RSK1/2 and MSK1/2 were determined by Western blotting from whole cell lysates; β-actin was used as the loading control. (**B**,**D**,**E**) Densitometric analysis of protein bands from three independent experiments for phospho-RSK1/2 in BT-549 cells (**B**), as well as phospho-RSK1/2 (**D**) and phospho-MSK1/2 (**E**) in MDA-MB-468 cells. (**F**,**G**) Kaplan–Meier (Km) plots for MSK1 (**F**) and MSK2 (**G**). Indicated in the KM plots is the relationship between high (red) or low (black) expression levels of the indicated genes and the survival of basal-like breast cancer patients, the hazard ratio (HR) and statistical significance. * denotes *p* < 0.05.

We next validated the expression and phosphorylation of the p90 ribosomal S6 kinase family proteins RSK1/2 and the related MSK1/2 in chemotherapy-resistant TNBC cell models by Western blotting. We show that RSK1/2 were expressed in BT-549 cells (Figure 6A) and in MDA-MB-468 cells (Figure 6C) and that phosphorylation of these proteins at S221/S227 in chemotherapy-resistant cells was both drug- and cell-type-dependent (Figure 6A–D). Expression of MSK1/2 on the other hand is restricted to the epithelial MDA-MB-468 cells and phosphorylation of MSK1/2 proteins at S376/360 was barely detected in DOX-R cells, slightly detected in PTX-R, and more intensely detected in CBP-R cells (Figure 6C,E).

Given that RSK1 and 2 as well as MSK1 and 2 proteins cannot be easily distinguished based on the molecular sizes or phosphorylation status, we again carried out KM Plotter analysis of the genes to identify the clinically relevant isoforms in basal-like breast cancer. This analysis revealed that reduced expression of RSK1 but not RSK2 (Figure 5H,I), and reduced expression of MSK2 but not MSK1 (Figure 6F,G), was associated with poor RFS of basal-like breast cancer patients. Together, this indicates that a subset specific proinflammatory cytokine signature, distinct regulation of GSK-3β activity, as well as the differential expression and activation of p90RSK proteins play a critical role in the maintenance of chemotherapy resistance of epithelial and mesenchymal-like cells in TNBC tumors.

## 3. Discussion

The goal of this study was to define the contribution of mesenchymal-like and epithelial TNBC cell types in TNBC tumors to chemotherapy resistance. The most salient findings were as follows: (1) typical TNBC tumors are predominantly epithelial tissues with discrete areas of mesenchymal-like TNBC cells, and that detection of Ki67 and AnxA6 together with EpCAM for epithelial/proliferative cells and vimentin for mesenchymal-like/invasive cells can be used to identify these cell types. (2) Resistance of TNBC cells to chemotherapy was associated with differential expression of cell type specific molecular drivers including downregulation of BIRC5 (survivin) and upregulation of CXCL8/IL-8 and M-CSF in mesenchymal-like cells, and upregulation of CTSS, MCP-1 and DKK-1 in epithelial TNBC cells. (3) Differential phosphorylation of GSK-3β at S9 was increased in chemotherapy-resistant epithelial cells but reduced in chemotherapy-resistant mesenchymal-like cells. (4) Chemotherapy resistance in epithelial cells, unlike in mesenchymal-like cells, was associated with activation of MSK1/2. Overall, this study highlights the complexity and heterogeneity of TNBC tumors and reveals that the diverse response to and frequent relapse of chemotherapy-residual TNBC may be attributed to the major differences in proinflammatory cytokine signaling and activation of downstream kinases. Based on these data, we propose that chemotherapy resistance in TNBC patients can be alleviated by targeting these factors or the associated pathways.

Mesenchymal-like TNBC cells are inherently resistant to chemotherapy while epithelial TNBC cells are generally sensitive to chemotherapy, but residual chemotherapy-resistant cells can acquire mesenchymal-like traits through epithelial-to-mesenchymal transition (EMT) that enhances their invasiveness and potential for metastasis [37]. Based on the Ki67 and AnxA6 expression status, BT-549 and MDA-MB-468 TNBC cells, as typical mesenchymal-like and epithelial cellular subsets respectively, indeed differed in terms of their proliferation rate, vimentin, Ki67 and AnxA6 expression and their response to chemotherapy agents. This study revealed differential cellular plasticity following prolong treatment of TNBC cells with chemotherapy agents. The drug-to-drug differences on the expression of AnxA6, Ki67, vimentin and EpCAM underscores the heterogeneity and cellular plasticity of TNBCs and the diverse responses to treatment [22].

Our findings that vimentin expression is significantly associated with Ki67 expression in TNBC patient tissues is consistent with the previously reported association of vimentin expression with high Ki67 expression and poor prognosis for TNBC patients [38]. Our data also confirms our previous report suggesting that the reciprocal expression of AnxA6 and Ki67 in TNBC tumor biopsies can delineate AnxA6-high/Ki67-low invasive TNBC cells from AnxA6-low/Ki67-high proliferative tumor cells [26]. The observed upregulation of vimentin in carboplatin and, to a lesser extent, paclitaxel-resistant mesenchymal-like TNBC cells and a reduction in the expression of EpCAM in carboplatin and paclitaxel-treated epithelial tumor cells are consistent with invasive tendencies for TNBC cells following treatment with chemotherapy agents [39].

The development of resistance to chemotherapy has long been associated with upregulation of oncogenes and downregulation of tumor suppressor genes [40,41], but also the modification of the tumor microenvironment to favor tumor growth and metastasis [42]. This has been extensively shown to help cancer cells evade apoptosis by mechanisms that include evasion of cellular stress, enhanced DNA repair, drug efflux, and activation of survival pathways [43].

Our study demonstrates that resistance of mesenchymal-like TNBC cells is associated with downregulation of the anti-apoptotic BIRC5 (survivin) and upregulation of CXCL8/IL-8 and M-CSF while that of epithelial tumor cells is associated with upregulation of CTSS, MCP-1 and DKK-1. These subset-specific molecular targets can block apoptosis, and signal via their cognate receptors to promote cell survival during chemotherapy. This is supported by previous studies showing increased expression of CXCL8/IL-8 in PTX resistance [44]. Increased secretion of macrophage colony-stimulating factor (M-CSF) is associated with poor prognosis in various cancer types, including breast cancer and promotes tumor invasion, metastasis and chemotherapy resistance [45]. Thus, upregulation of CXCL8/IL8 and M-CSF and downregulation of survivin, an inhibitor of apoptosis, clearly demonstrates the promotion of the survival of residual chemotherapy-resistant mesenchymal-like TNBC cells.

The chemokine CCL2/MCP-1, has been shown to promote breast cancer progression and metastasis to lungs and bone in mouse models of breast cancer [46,47]. Cathepsin S (CTSS) has been shown to be upregulated in basal-like 1 TNBC molecular subtype with defects in DNA damage repair pathways [28] and may promote resistance to chemotherapy by facilitating the degradation of BRCA1 [48,49]. Dickkopf-1 (DKK1) is a secreted protein that promotes chemotherapy resistance by inhibiting cancer cell migration and invasion via the Wnt/β-catenin signaling pathway [50,51]. Therefore, the survival and subsequent progression of chemotherapy-resistant epithelial TNBC cells is at least in part promoted by DKK1, MCP-1 and the protease CTSS via inhibition of DNA repair, modulation of immune cell infiltration and proinflammatory cytokine survival pathways [52,53,54].

Although GSK 3β has been shown to promote chemotherapy and radiotherapy resistance as treatment options for tumors [55], it has been shown to be a tumor suppressor in some cancers including breast cancer and a tumor promoter in other types of cancers [56]. The reduced inhibitory phosphorylation of GSK 3β (p-S9) in PTX-R mesenchymal-like BT-549 cells and increased GSK 3β (p-S9) in PTX-R epithelial MDA-MB-468 cells indicates significant differences in the maintenance of chemotherapy resistance in these TNBC cell types [57]. This, at least in part, suggests stabilization of β-catenin in epithelial cells and degradation of β-catenin in mesenchymal-like cells. Given that the expression of b-catenin in the array did not change with chemotherapy resistance, it is possible that the GSK3β activation status influences other survival pathways mediated via mTORC1 and NF-kB [58]

Anthracyclines and platinum drugs are known to induce DNA damage which adversely affects cell cycle progression and induce apoptosis. Taxanes on the other hand affect microtubule polymerization and together can lead to serious cellular stress, disrupt protein synthesis and metabolic processes. Detection of phosphorylated mitogen- and stress-activated kinases 1/2 (MSK1/2) in MDA-MB-468 cells and phosphorylated 90 kDa ribosomal S6 kinase (RSK1/2) in both BT-549 and MDA-MB-468 cells suggest the activation of these proteins in response to treatment-induced stress [59]. Given that these proteins are very similar and can act in redundant pathways, our KM Plotter database analysis together with Western blotting confirmed that RSK1 and MSK2 are the relevant chemotherapy responsive, druggable serine/threonine RPS6KA kinase family targets in TNBC cells. Interestingly, the phosphorylation of RSK1/2 was similarly affected in the chemotherapy-resistant mesenchymal-like and epithelial TNBC cells, while MSK1/2 are specifically expressed in epithelial cells and activated by prolonged treatment with PTX and even more so with CBP. MSK1/2 are nuclear protein kinases that are phosphorylated and activated by the MAPK/ERK pathway [60] and can promote or suppress different genes at multiple levels, which can result in the promotion or prevention of cancer metastasis [60,61]. Combining standard-of-care chemotherapy agents with pharmacological RSK1/MSK2 targeting agents such as APIO-EE-07 [62], SL0101 analog [63] and other commercially available inhibitors may provide viable options to relieve chemotherapy resistance in TNBC.

## 4. Materials and Methods

### 4.1. Cell Culture

The mesenchymal-like BT-549 cells (ATCC, HTB-122) and epithelial-like MDA-MB-468 (ATCC, HTB-132) cells were obtained from the American Type Culture Collection (Manassas, VA, USA). Only the early (<5) passages were used in experiments. BT-549 cells were cultured in DMEM/F12 (Thermo Fisher Scientific, Waltham, MA, USA) medium supplemented with 10% fetal bovine serum (FBS), 5% sodium bicarbonate and 1% Penicillin/Streptomycin. MDA-MB-468 cells were cultured in Leibovitz’s L-15 medium (Thermo Fisher Scientific, Waltham, MA, USA) supplemented with 10% fetal bovine serum (FBS), 5% sodium bicarbonate and 1% Penicillin/Streptomycin. Cells were maintained at 37 °C with 5% CO_2_ in a humidified incubator. Media were changed every 2–3 days and cells were passaged by using TrypLE cell dissociation reagent (Gibco, Grand Island, NY, USA). The cells were regularly checked for mycoplasma contamination using mycoplasma detection kit from InvoivoGen US (San Diego, CA, USA).

### 4.2. Establishment of Resistant TNBC Cell Lines

Doxorubicin HCl (DOX) (NSC 123127), Paclitaxel (PTX) (NSC 125973), and Carboplatin (CBP) (NSC 241240) were procured from Selleck Chemicals LLC (Houston, TX, USA) and used to establish drug-resistant BT-549 and MDA-MB-468 cells using the pulse exposure and stepwise dose escalation method as previously described for cisplatin-resistant ovarian cancer cells [64]. Briefly, the cells were treated with each chemotherapy agent by exposure to gradually increasing concentrations of the drugs over a period of 6 months. BT-549 or MDA-MB-468 cells were cultured in the drug-containing media for 48 h, allowed to recover in drug-free media until 80% confluency, and the treatment cycle repeated with the same concentration of drug. A new cycle began with maintaining the cells in media containing a slightly higher concentration of the drugs and the process continued until concentrations at which the cells could not survive due to cytotoxicity.

### 4.3. Cell Proliferation

Cells were seeded at a density of 5000 cells per well in an 8-well chamber Millicell EZ slide (Sigma-Aldrich, St. Louis, MO, USA) for 48 h. Cells were stained using the Baseclick GmbH EdU HTS assay kit as described by the manufacturer (Sigma-Aldrich, St. Louis, MO, USA). Briefly, cells were incubated with 10 µM EdU for 3 h, then fixed and permeabilized in cold methanol at −20 °C followed by EdU detection using the red fluorescent Cyanine 5 (Cy5) dye and cell imaging (546/579 nm Ex/Em).

### 4.4. Cell Viability and 3D Clonogenic Assays

For cell viability assays, cells were seeded in 96-well plates at a density of 5000 cells per well and cultured overnight in complete medium. Cells were subsequently treated with the indicated drugs for 72 h and then incubated with diluted PrestoBlue™ Cell Viability Reagent (Invitrogen, Carlsbad, CA, USA) for 1 h at 37 °C in a humidified incubator. The viability of the cells was measured by fluorescence intensity (530/590 Ex/Em) by using a BioTek Synergy HT Microplate Reader (Hampton, NH, USA). For 3D clonogenic assays, cells were plates in 24 well ultra-low attachment plates in complete medium supplemented with 2% Matrigel and the indicated drugs. Cells were cultured for up to 10 days and brightfield images were captured using Accu-Scope EXI-410 inverted phase contrast microscope (Commack, NY, USA) at 10× magnification.

### 4.5. Immunofluorescence

Cells were seeded at a density of 5000 cells per well in 8-well chamber Millicell EZ slide (Sigma-Aldrich, St. Louis, MO, USA) for 48 h and immunofluorescence was carried out as previously described [32]. Cells were fixed with cold methanol at room temperature (RT) for 20 min, followed by blocking with 1% bovine serum albumin (BSA) in phosphate-buffered saline (PBS) for 1 h. Cells were then incubated for 1 h with the following primary antibodies Annexin VI (Santa Cruz Biotechnology, Dallas, TX, USA), Vimentin (Santa Cruz Biotechnology, Dallas, TX, USA) and Ki67/MKI67 (R&D Systems, Inc., Minneapolis, MN, USA). Cells were then stained using Alexa Fluor™ Plus 488 conjugated Donkey anti-Rabbit IgG (Invitrogen, USA) and Alexa Fluor™ 568 conjugated Donkey anti-Mouse IgG (Invitrogen, USA) for 45 min and counterstained with ProLong™ Gold Antifade mounting fluid with DAPI (Invitrogen) for 10 min, at RT. Imaging was performed using BZ-X710 All-in-One Fluorescence Microscope (Keyence, Itasca, IL, USA).

### 4.6. Immunocytochemistry and Multiplex Fluorescent Immunohistochemistry

The use of clinical and pathological tissues from 22 TNBC patients in this study was approved by the Meharry Medical College Institutional Review Board (IRB) as exempt research. Slides containing formalin fixed paraffin embedded deidentified biopsies (primary treatment naïve) or residual chemotherapy-resistant tumor tissues were obtained from the Vanderbilt Translational Pathology Shared Resource as previously described [26]. The slides were processed for standard immunohistochemistry (IHC) and images were captured by using Nikon A1R confocal laser scanning microscope and NIS-Elements software version 6.10.01 (Nikon Inc., Melville, NY, USA).

Multiplex fluorescent IHC was performed by using the tyramide signal amplification procedure as previously described [65]. Deparaffinized tissues were incubated in citrate buffer for antigen retrieval, then permeabilized and blocked with 3% hydrogen peroxide solution at 37 °C for 20 min. Non-specific blocking with normal goat serum (Santa Cruz Biotechnology) was performed, followed by sequential incubation with the following primary antibodies: Annexin VI (Santa Cruz Biotechnology, Dallas, TX, USA), Vimentin (Santa Cruz Biotechnology, Dallas, TX, USA) and Ki67/MKI67 (R&D Systems, Inc., Minneapolis, MN, USA). Incubation with each primary, and the corresponding secondary, antibodies was followed by the antigen retrieval step to remove unbound antibodies. After secondary antibody incubation, tissue slides were washed and then incubated with a tyramide-conjugated fluorescent dye (Ty-fluor), rinsed and mounted with coverslip in mounting media containing DAPI for nuclear counterstaining.

Whole slide imaging and analysis was performed at the Digital Histology Shared Resource at Vanderbilt University Medical Center. The fluorescent, immunostained tissue slides were imaged on an Aperio Versa 200 automated slide scanner (Leica Biosystems, Wetzlar, Germany) at 20× magnification to a resolution of 0.323 µm/pixel. Tumor areas were mapped using Ariol Review software version V6.0.4 and the staining intensity of the images was performed using the Ariol software capable of triple- and quadruple-stained immunohistochemical (IHC) quantification.

### 4.7. Proteome Profiler Antibody Arrays

The levels of 84 cancer-related proteins in cell lysates prepared from control and chemotherapy agent-resistant cells were assayed by using the Human XL Oncology Array kit (R&D Systems; cat. no. ARY026). The activation status of 37 kinases was also assessed using the Human Phospho-Kinase Array Kit (R&D Systems; cat. no. ARY003C). Nitrocellulose membranes spotted in duplicate with capture antibodies against cancer-related proteins or phospho-kinases were incubated with cell lysates prepared from control and chemotherapy drug-resistant cells and processed as recommended by the manufacturer (R&D Systems, Minneapolis, MN, USA). The blots were developed using enhanced chemiluminescent reagent (Perkin Elmer, Springfield, IL, USA), and the images were acquired with the ChemiDoc™ Touch imaging system (Bio-Rad, Hercules, CA, USA). Densitometric analysis of the arrays was carried out by using the ImageJ version 1.54d particle analysis module. The densitometric values from the duplicate spots representing each protein were normalized to the array controls in each membrane and the differences in the detected proteins between the control and chemotherapy agent-resistant cells were expressed as fold changes (FC). Proteins with FC > 1 were considered to be upregulated while those with FC < 1 were considered to be downregulated.

### 4.8. Western Blotting

Cells were cultured until ~80% confluency, washed twice and then scraped in ice-cold Hanks’ balanced salt solution (HBSS) containing 1 mM Ca^2+^ and 1 mM Mg^2+^. Cell pellets were resuspended in radioimmunoprecipitation assay (RIPA) lysis buffer (50 mM Tris-HCl, pH 7.4, 1% NP-40, 0.1% sodium deoxycholate, 150 mM NaCl, 1 mM EDTA) containing freshly added protease and phosphatase inhibitors, incubated on ice for 30 min and centrifuged at >10,000× *g* for 10 min at 4 °C. Western blotting was carried out as previously described [33,66], using primary antibodies against Annexin VI (AnxA6), Vimentin and Ep-CAM (Santa Cruz Biotechnology, Dallas, TX, USA); total and phosphorylated RSK1/2 and MSK1/2 (Cell Signaling Technology, Danvers, MA, USA) and β-actin (Santa Cruz Biotechnology, Dallas, TX, USA) was used as the loading control. The blots were revealed with enhanced chemiluminescent reagent (Perkin Elmer), imaged with the ChemiDoc™ Touch gel imaging system (Bio-Rad, Hercules, CA, USA) and quantified by densitometry using ImageJ software.

### 4.9. Statistical Analysis

Statistical analysis was performed using GraphPad Prism version 9.5.1 (San Diego, CA, USA). For all other analyses, paired t-tests were used and a *p*-value < 0.05 was considered statistically significant.

## 5. Conclusions

This study highlights the complexity and heterogeneity of TNBC tumors and emphasizes the distinct characteristics and responses of mesenchymal-like and epithelial TNBC cells to chemotherapy. Chemotherapy resistance of epithelial and mesenchymal-like TNBC cellular subsets is not only associated with distinct profiles of proinflammatory and immune cell chemotactic cytokines but also modulates the activities of GSK-3β, p90 RSK1/2 and the related MSK1/2. Targeting these factors and/or the associated signaling pathways may help overcome chemotherapy resistance in TNBC.

Limitations of this study include the following: (1) The small sample size of chemotherapy-treated samples with the corresponding residual tumors. Further examination of the expression of some of the identified factors in larger cohorts of diverse untreated biopsies and residual treatment-resistant tumors may validate their role in chemotherapy resistance. (2) With our current knowledge of molecular and cellular heterogeneity of TNBC, the validation of our findings including the identified cytokines and phosphoproteins in multiple cell lines, patient-derived organoids, in vivo TNBC models (e.g., xenograft or patient-derived tumor models) and/or a larger sample size of chemotherapy-resistant tumor tissues is warranted. However, this may be complicated by the differential effects of distinct chemotherapy agents on phenotypically distinct cell types as demonstrated in this study. (3) The antibody arrays as discovery tools do not provide a comprehensive picture of factors that mediate chemotherapy resistance. Complementing these assays with genome-wide proteomic and transcriptomic profiling may provide more robust data on cellular subset specific molecular drivers of chemotherapy resistance. The caveat to the ensuing big data is the requirement for additional unbiased gene/protein stratification criteria to identify the most likely cellular subtype specific drivers of chemotherapy resistance. Our future studies are geared towards addressing some of these key avenues for future studies.

## Figures and Tables

**Table 1:** Half-maximal inhibitory concentrations of standard chemotherapy drugs in parental and drug-resistant TNBC cells.

Chemotherapy Agents	Half-Maximal Inhibitory Concentrations (IC_50_) (µM)			
	BT-549-Par	BT-549-R	Drug Fold Resistance	*p*-Value
Doxorubicin	0.15	0.24	1.60	0.01 *
Paclitaxel	0.013	0.014	1.08	0.3391
carboplatin	148.5	336.0	2.26	0.0311 *
				
	**MDA-MB-468-Par**	**MDA-MB-468-R**	**Drug Fold Resistance**	***p*** **-Value**
Doxorubicin	0.02	0.07	3.5	0.0388 *
Paclitaxel	0.06	0.21	3.5	0.0002 *
carboplatin	50.29	143.3	2.85	0.0731

* denotes statistically significant compared to parental control cells.

## Data Availability

The original contributions presented in this study are included in the article/Supplementary Materials. Further inquiries can be directed to the corresponding author.

## Supplementary Materials

The following supporting information can be downloaded at: https://www.mdpi.com/article/10.3390/ijms27073157/s1.
